# Clinical and bacteriological profile of diabetic foot infections in a tertiary care

**DOI:** 10.1186/s13047-020-00406-y

**Published:** 2020-06-16

**Authors:** Teik Chiang Goh, Mohd Yazid Bajuri, Sivapathasundaram C. Nadarajah, Abdul Halim Abdul Rashid, Suhaila Baharuddin, Kamarul Syariza Zamri

**Affiliations:** 1 Department of Orthopaedic and Traumatology, Universiti Kebangsaan Malaysia Medical Centre, Hospital Canselor Tunku Muhriz, 56000 Cheras, Malaysia; 2 Department of Orthopaedic and Traumatology, Hospital Melaka, 75400 Melaka, Malaysia; 3 Department of Microbiology, Hospital Melaka, 75400 Melaka, Malaysia

**Keywords:** Bacteriology profile, Diabetic foot infections, Microbial sensitivity tests, Retrospective studies

## Abstract

**Background:**

Diabetic foot infection is a worldwide health problem is commonly encountered in daily practice. This study was conducted to identify the microbiological profile and antibiotic sensitivity patterns of causative agents identified from diabetic foot infections (DFIs). In addition, the assessment included probable risk factors contributing to infection of ulcers that harbour multidrug-resistant organisms (MDROs) and their outcomes.

**Methods:**

We carried out a prospective analysis based on the DFI samples collected from 2016 till 2018. Specimens were cultured with optimal techniques in addition to antibiotic susceptibility based on recommendations from The Clinical and Laboratory Standards Institute (CLSI). A total of 1040 pathogens were isolated with an average of 1.9 pathogens per lesion in 550 patients who were identified with having DFIs during this interval.

**Results:**

A higher percentage of Gram-negative pathogens (54%) were identified as compared with Gram-positive pathogens (33%) or anaerobes (12%). A total of 85% of the patients were found to have polymicrobial infections. *Pseudomonas aeruginosa* (19%), *Staphylococcus aureus* (11%) and Bacteroides species (8%) appeared to be the predominant organisms isolated. In the management of Gram-positive bacteria, the most efficacious treatment was seen with the use of Vancomycin, while Imipenem and Amikacin proved to be effective in the treatment of Gram-negative bacteria.

**Conclusion:**

DFI’s are common among Malaysians with diabetes, with a majority of cases displaying polymicrobial aetiology with multi-drug resistant isolates. The data obtained from this study will be valuable in aiding future empirical treatment guidelines in the treatment of DFIs. This study investigated the microbiology of DFIs and their resistance to antibiotics in patients with DFIs that were managed at a Tertiary Care Centre in Malaysia.

## Background

A worrying global health predicament in the modern age is Diabetes Mellitus (DM). Approximately 150–170 million of the world’s population is suffering from this condition and its prevalence will most likely multiply by two folds by 2025 as per the World Health Organization (WHO) reports [1]. The WHO has identified the leading 10 countries in Asia with a large number of people with DM including India, Japan, China, Korea, Bangladesh, Thailand, Indonesia, Philippines and Malaysia. Malaysia is forecast to have an estimated leap from 2.5 million people with DM in the year 2018 to a drastic and frightening increase to 5 million people by the year 2030 [2]. The preponderance of DM in Malaysia is almost 32% of the population which has significantly increased from 0.6% in 1960 to 2.1% in 1982, 6.3% in 1986, 8.3% in 1996 and 14% in 1998 [3, 4]. DM is now estimated to affect the lives of 16–18% of Malaysians [3, 5]. Patients who do not have good control of their DM are at risk of developing complications arising from diabetes which are macrovascular and microvascular. Among these complications are neuropathy, retinopathy and pedal ulcers with or without gangrene [3]. Almost 15% of patients with DM eventually end up developing foot ulcers that can progress to osteomyelitis [6]. The progression of these wounds from superficial to debilitating infections are facilitated by impaired defence mechanisms of the body and delayed treatment. The subcutaneous tissue infections have the ability to spread to the deeper structures which will ultimately progress into complications including gangrenous changes and also amputations [7]. Poor control of DM puts a patient at higher risk of skin infections as being in a state of hyperglycaemia impairs the efficiency of the body’s immune or defence mechanism [8]. Diabetic foot infections (DFIs) are complex, rampant and costly ramifications of DM. These infections account for the majority of DM related hospitalisations other than non-traumatic amputations [9]. Few have studied the relationship between number of organisms identified and the types of infections that occur from it. In 2003, Frykberg concluded that most mild infections are commonly monomicrobial in which Gram-positive cocci such as Streptococcus species and *Staphylococcus aureus* were identified.

Contrarily, the more severe forms of DFI’s are commonly polymicrobial in which the causative organism include Gram-negative bacilli (e.g., Klebsiella species., Proteus species., *Escherichia coli*, Pseudomonas species.) aerobic Gram-positive cocci, and anaerobes [10].

Prompt initiation of ideal therapy for DFIs plays a role in the reduction of morbidities interlinked with infections. Despite that, there can be a reduction in the number of hospitalisations and their durations in addition to reducing the incidence of major limb amputation. There are many factors that contribute to successful clinical outcomes, which include prompt lesion recognition followed by host factor modification, expeditious commencement of ideal antibiotic therapy plus efficient surgical intervention which includes debridement of bone and soft necrotic tissue. Prompt antibiotic therapy should be initiated to improve limb saving probabilities as many DFIs are true emergencies [11]. The aim of this study was to further enhance understanding on the bacteriology of diabetic foot ulcers alongside the assessment of the in vitro antimicrobial susceptibility of the offending pathogens in the General Hospital of Melaka, which is located in the historical state of Melaka, Malaysia and Universiti Kebangsaan Malaysia Medical Centre which is located in central Kuala Lumpur, both with a capacity to accommodate nearly one thousand patients. These two hospitals are major general tertiary care centres in their respective states and are the primary centres that receive referrals for further management of DFI’s.

## Materials and methods

This prospective study included 550 patients with longstanding and newly diagnosed DM who presented with lower extremity infections requiring admission to the General Hospital of Melaka and Universiti Kebangsaan Malaysia Medical Centre. This study was conducted over a period of 2 years between 1st February 2016 and 1st February 2018. There was no duplications in the recruitment of patients in the study. Patients underwent clinical assessment and proper grading of their foot lesions was carried out in accordance with the DFI severity classification systems including the Wagner Classification System and the University of Texas Wound Classification System [12]. The classifications of wounds were according to the severity which was mild, moderate or severe based on the size, depth and level of the tissue involved in the infection, presence of metabolic instability or any systemic manifestations of infection. Briefings were given to patients regarding the details of the study. Patient information including age, gender, type of diabetes and duration of DFI’s were recorded. Various specimens (wound exudates, pus or tissue biopsy) from the ulcerated regions were collected for microbiological studies. The surface of those regions was thoroughly rinsed with sterile normal saline solution and pus samples were taken using sterile cotton swabs. A total of 550 patients were identified from request forms and their clinical specimens were subsequently dispatched to the microbiology laboratory for further analysis. The forms were designed to provide pertinent demographic data and clinical information including a patient’s age, gender, race, nature of the specimen, examination required, diagnosis and details of antimicrobial therapy. The request forms were completed and signed by the respective treating medical officers. Data were recorded according to patient’s age, gender, ethnicity alongside nature of the clinical specimen, species of the isolated pathogen and antibiogram of the clinical isolates.

### Wagner’s classification of diabetic foot ulcers

Grade 0: no ulcer in a high-risk foot.

Grade 1: superficial ulcer involving the full skin thickness but not underlying tissues.

Grade 2: deep ulcer, penetrating down to ligaments and muscles, but no bone involvement or abscess formation.

Grade 3: deep ulcer with cellulitis or abscess formation, often with osteomyelitis.

Grade 4: localized gangrene.

Grade 5: extensive gangrene involving the whole foot.

### University of Texas Wound Classification System of diabetic foot ulcers

Grade IA: not infected, no ischemic superficial ulceration.

Grade IB: infected, no ischemic superficial ulceration.

Grade IC: ischemic, no infected superficial ulceration.

Grade ID: ischemic, infected superficial ulceration.

Grade IIA: not infected, no ischemic ulcer that penetrates to capsule or bone.

Grade IIB: infected, no ischemic ulcer that penetrates to capsule or bone.

Grade IIC: ischemic, no infected ulcer that penetrates to capsule or bone.

Laboratory investigations and hospital records of patients recruited into the study between February 2016 to February 2018 were reviewed meticulously. The technique of specimen collection included clinically infected wounds while excluding superficial or colonizing organisms. Specimen isolates were not duplicated in this study. The specimen cultures were obtained once the wound surface had been irrigated with copious normal saline, and we subsequently debrided the superficial tissue and separated them from the exudates to isolate the colonizing pathogenic flora. Specimens were obtained by curetting the ulcer base post debridement and by aspirating the infected skin or deep tissues. The specimens underwent gram staining and inoculation on both selective and non-selective media including MacConkey agar (Oxoid), blood agar (BA; Oxoid, Basingstoke, UK), chocolate agar and 5% (v/v) BA supplemented with vitamin K1 (1 μg/ml), gentamicin (75 μg/ml) (GBA) and haemin (5 μg/ml). The inoculated plates were incubated under conducive atmospheric conditions for 24–48 h to enable bacterial isolation. Isolated organisms were identified by traditional microbiological methods: Analytical profile index 20 (API 20) Strep for streptococci and enterococci, API 20A (bioMérieux, Marcy l’Etoile, France) and GLC (Chromopak, CP9001, The Netherlands) for anaerobes, API 20E for Gram-negative aerobes and API Staph for staphylococci. According to the standardised procedures described by Sharma, the bacteria were isolated and classified by microscopy, morphology and biochemical testing [13].

### Antimicrobial susceptibility testing

In accordance with the guidelines of the Clinical and Laboratory Standards Institute (CLSI), confirmation of antimicrobial susceptibility of the bacterial isolates was carried out by using the disk diffusion method [14]. Isolated bacterial colonies were suspended in sterile distilled water and matched to the 0.5 McFarland standard [5, 15]. A sterile swab was soaked into the inoculums and subsequently used to streak the Mueller–Hinton agar plates for Gram-negative bacilli, Staphylococci, and Enterococci. For Streptococci, the Mueller–Hinton agar was supplemented with 5% horse blood as described in the CLSI guidelines and followed by application of antibiotic discs on the plates. The inoculated agar plates were incubated at 35–37 °C for 18–20 h. Then, the diameter of the inhibition zone was interpreted based on the guideline suggested by the CLSI. Staphylococcus aureus was tested for Methicillin resistance using a 1 μg Oxacillin disc. Reference strains of *Escherichia coli* and *Pseudomonas aeruginosa* were used as a control for Gram-negative bacteria and were included in the tests. *Staphylococcus aureus* and *Enterococcus faecalis* were used as Gram-positive control strains [14].

## Results

### Demographic characteristics

All 550 patients successfully recruited into the study were recruited. All had been diagnosed with Diabetic Foot Ulcer to participate in this particular study. The data of these patients were compiled and summarized in Table 1. In this study, 337 out of 550 (61%) were male and 213 out of 550 (39%) were female with a male-to-female ratio of 1.58:1. The patients’ age ranged from 37 to 91 years old with a mean of 56.7 years. Out of the total population, only 10 (1.8%) patients presented with type 1 DM and the rest 540 (98.2%) were type 2 DM. The majority of patients were Malaysian nationals 544 (98.9%). Indians and Malays accounted for 40% (*n* = 220) and 39% (*n* = 214) respectively, while the Chinese population accounted for 20% (*n* = 110) followed by 1% (*n* = 6) of the others.

**Table 1:** Demographic distribution of 550 patients with diabetic foot infections

Age (years)	Malaysian (***n*** = 544)		Non-Malaysian (***n*** = 6)		Total no. (%) (***n*** = 550)
	M	F	M	F	
40≤	27	26	0	1	54 (9.8)
41–50	65	46	3	2	116 (21)
51–60	137	55	0	0	192 (35)
61–70	85	67	0	0	152 (27.6)
71–80	18	15	0	0	33 (6)
81–90	2	0	0	0	2 (0.4)
≥91	0	1	0	0	1 (0.2)
Total	334	210	3	3	550 (100)

### Microbiology

From 550 patients, we were able to isolate 1040 pathogens averaging at 1.8 per lesion. We included the specimens with clinically significant pathogens which were wound tissue (220; 40%), swabs (220; 40%), pus (83; 15%) and bone (27; 5%). Patients were graded based on foot lesions and the number of bacterial isolates. Their numbers are shown in Table 2. Table 3 presents the multiple organisms isolated from the DFIs. 54% of aerobic gram-negative bacteria were isolated and it was the most commonly isolated pathogens. The four most common microbial isolates were *Pseudomonas aeruginosa* (19%), *Staphylococcus aureus* (11%), Methicillin-resistant *Staphylococcus aureus* (8%) and Bacteroides species (spp.) (8%).

**Table 2:** The number of patients in each grade of diabetic foot infection and the total number of bacteria isolated from different grades of diabetic foot infection

Grades of diabetic foot infection	No. of patients	A positive culture for bacteria		
		1	2	> 2
Mild	203	30	51	122
Moderate	227	34	57	136
Severe	120	18	30	72
Total	550	82 (15%)	138 (25%)	330 (60%)

**Table 3:** The proportion and frequency of microbial isolation from the samples

Bacteria	Total number	Proportion	Frequency
	***N*** = 1040		
**Gram-positive bacteria**	**348**	**33**	**63**
***Staphylococcus aureus***	**116**	**11**	**21**
**Methicillin-resistant*S. aureus***	**86**	**8**	**15**
Enterococcus spp.	63	6	11
Group B streptococci	47	4	9
Other streptococci	27	3	5
Other Staphylococcus spp.	9	1	2
**Gram-negative bacteria**	**558**	**54**	**100**
***Pseudomonas aeruginosa***	**192**	**19**	**35**
*Proteus mirabilis*	74	7	13
Klebsiella pneumonia	71	7	13
Enterobacter spp.	69	6	12
*Escherichia coli*	60	6	10
Non-fermenters	29	3	5
Morgenella spp.	20	2	4
Other Gram-negative	16	2	3
Serratia marcescens	15	1	3
Citrobacter spp.	12	1	2
**Anaerobes**	**123**	**12**	**22**
**Bacteroides spp.**	**84**	**8**	**15**
Anaerobic Streptococci	18	2	3
Microaerop Streptococci	10	1	2
Candida spp.	9	1	2
Clostridium spp.	6	0.5	1
Fusobacterium spp.	5	0.5	1

Proportion = the percentage of isolates organism divided into the total number of isolated organisms.

Frequency = the percentage of the organisms divided into the total number of patients.

### Antibiotic susceptibility

Analysis of this study showed that 468 (85%) of the 550 infected patients had multiple microorganism infections. The most common microbial isolates in Gram-positive bacteria were Staphylococcus aureus (11%), Gram-negative bacteria was Pseudomonas aeruginosa (19%) and Anaerobes was Bacteroides species (8%) respectively.

Table 4 illustrates the antimicrobial susceptibility pattern of the Gram-positive cocci. Out of the 150 Staphylococcus aureus isolates, 94.8, 62.0, and 56% were penicillin-resistant, tetracycline and erythromycin, respectively. Vancomycin, the most sensitive antibiotic for Staphylococcus aureus organism. The Enterococcus spp. were susceptible to vancomycin and tetracycline, however, 63.5 and 15.9% were resistant to erythromycin and penicillin, respectively. The Group B Streptococcus isolates were susceptible to co-amoxiclav, penicillin, and ampicillin, but 51% were resistant to tetracycline. Table 5 demonstrates the pattern of antibiotic resistance for Gram-negative bacilli. Imipenem and Amikacin were the most active antimicrobial agents tested against all of the microbial isolates. Pseudomonas aeruginosa resistant towards Ciprofloxacin and Piperacillin-Tazobactam, both 18% resistance. As for Proteus species and Klebsiella pneumonia, both were highly resistant towards Ampicillin (45% & 79% respectively). The antibiotic susceptibility pattern of isolated anaerobes is shown in Table 6. Amoxicillin, Ampicillin, Cefoxitin, Metronidazole and Piperacillin/Tazobactam showed excellent sensitivity against all anaerobes; there were no resistance towards these antibiotics. Nonetheless, 67% of the anaerobic streptococci and 32% of the Bacteroides species were resistant towards clindamycin.

**Table 4:** Resistance pattern for most common Gram-positive cocci towards antimicrobials

Antimicrobial agents	Gram-positive organisms (% resistant)					
	S. aureus		Enterococcus spp.		Group B Streptococcus	
	***n*** = 116	(%)	***n*** = 63	(%)	***n*** = 47	(%)
Ampicillin	59	50.8	–	–	0	0.0
Clindamycin	56	48.2	–	–	5	10.6
Cloxacillin	60	51.7	–	–	–	–
Co-amoxiclav	59	50.8	–	–	0	0.0
Erythromycin	65	56.0	40	63.5	8	17.0
Fusidic acid	60	51.7	–	–	–	–
Gentamicin	52	44.8	1	1.6	2	4.3
Penicillin	110	94.8	10	15.9	0	0.0
Rifampicin	1	0.8	–	–	–	–
Sulfamethoxazole-Trimethoprim	30	25.8	–	–	–	–
Tetracycline	72	62.0	0	0.0	24	51.0
Vancomycin	0	0.0	0	0.0	0	0.0

**Table 5:** Resistance pattern of most common Gram-negative organisms towards antimicrobials

Antimicrobial agents	Gram-negative organisms (% resistant)					
	***P. aeruginosa***		Proteus spp.		K. pneumonia	
	***n*** = 192	(%)	***n*** = 74	(%)	***n*** = 71	(%)
Amikacin	15	8	0	0	0	0
Amoxicillin clavulanic acid	22	11	17	23	13	18
Ampicillin	–	–	33	45	56	79
Cefotaxime	–	–	0	0	5	7
Ceftazidime	20	10	–	–	–	–
Cefuroxime	2	1	6	8	6	8
Ciprofloxacin	34	18	12	16	10	14
Gentamicin	16	8	13	18	8	11
Imipenem	17	9	0	0	0	0
Piperacillin	17	9	10	14	27	38
Piperacillin-Tazobactam	34	18	0	0	3	4
Sulphonamides Trimethoprim	–	–	17	23	–	–

**Table 6:** Susceptibility pattern of most common anaerobic bacterial isolates towards antimicrobials

Antimicrobial agents	Proportion susceptible (%)							
	Anaerobic Streptococci		Bacteroides species		Clostridium species		Fusobacterium species	
	***n*** = 18	(%)	***n*** = 84	(%)	***n*** = 6	(%)	***n*** = 5	(%)
Amoxicillin	–	–	–	–	–	–	–	–
Ampicillin/CA	18	100	84	100	6	100	5	100
Erythromycin	18	100	25	30	–	–	–	–
Cefoxitin	18	100	84	100	6	100	–	–
Clindamycin	12	67	27	32	–	–	–	–
Metronidazole	18	100	84	100	6	100	5	100
Penicillin-G	18	100	3	4	6	100	–	–
Piperacillin	18	100	75	89	6	100	–	–
Piperacillin/tazobactam	18	100	84	100	6	100	5	100
Tetracycline	–	–	–	–	–	–	–	–
Vancomycin	–	–	–	–	–	–	–	–

## Discussion

DFIs are a burgeoning predicament in Malaysia owing to the high incidence of DM among Malaysians where 1/5th of the population aged more than 30 years old were diagnosed with DM Type 2 reported by Bajuri et al. [16]. Our particular study features significant elements including the etiological agents of DFIs which are predominantly polymicrobial and contain both Gram-positive and Gram-negative bacteria. Anaerobes contribute to the infection by approximately 0.25 anaerobic bacteria per culture-positive specimen, with the majority of the aetiological agents being multi-resistant. When detected in DFI’s, anaerobes are commonly isolated from cultures that concurrently harbour aerobes. Notably, the isolation rate of anaerobes was lower than expected in our study despite its prevalence, especially in comparison with a report by Goldstein et al. [17]. There are many factors that may have contributed to this outcome which include suboptimal sampling or transportation of samples in addition to the use of inappropriate mediums. Previous studies stated that DFI’s predominantly and usually harbour Gram-positive aerobes [18]. On the contrary, most of our patient’s, the major infective organisms were gram-negative bacteria [19].

We discovered that the most common single pathogen isolation in diabetic foot infections was S. Aureus [11, 20, 21]. The prevalence of S. Aureus was 76 and 78% in studies according to Goldstein et al. and Kajetan et al. [21–23]. However, we have reported a much lower prevalence when compared with earlier reports. This contrast is to be expected as the majority of our patients had severe forms of DFIs which are commonly polymicrobial when compared with mild DFIs that are usually associated with aerobic Gram-positive cocci, such as S. Aureus. Unfortunately, for the past two decades, MRSA has been a pathogen of concern especially with the evolution of community-acquired MRSA [13]. Our study concluded that the MRSA isolation rate increased to nearly four-folds when compared with previous Malaysian studies [5]. This development is likely due to a lack of strict antibiotic prescription guidelines alongside the lack of adherence to infection control measures in the hospital with a higher prevalence of MRSA in the community. If not rectified, these practices will likely alter empirical antimicrobial therapy.

In addition, approximately 25% of our patients demonstrated Streptococci cultures with a large number being *S. agalactiae* which is also known as group B Streptococcus [24]. This finding came as no surprise as *S. agalactiae* is a commonly identified DFI pathogen [17, 18]. Enterococci are considered to be commensals and exhibit low virulence, excluding those who are in an immunocompromised state such as those with DM in which commensals may also become opportunistic pathogens. Despite that, based on previous reports by various investigators, a large number of our patients’ DFIs were caused by other different organisms [17].

Previous antimicrobial use, prolonged hospitalizations, chronic wounds, and surgical interventions which are all distinctive of patients with this type of infection in our hospital are likely contributors to the comparatively large prevalence of P. Aeruginosa in this study. Moreover, a high proportion of E.Coli and Klebsiella spp. isolates were positive for ESBLs and comparison with studies in other regions deduced that the incidence of ESBL-producing Klebsiella spp. and *E. coli* was higher in our population of patients with DFIs [5]. Hence, routine screening towards ESBL-producing Enterobacteriaceae should be emphasised.

Piperacillin–Tazobactam, Amikacin and Imipenem were the greatest advantageous antimicrobials against aerobic Gram-negative bacteria. As for aerobic Gram-positive cocci and anaerobes, the most effective antimicrobials where Vancomycin and Metronidazole respectively [25]. It is vital to ensure meticulous and thoughtful choices of antimicrobial therapy guided by culture findings in addition to the antimicrobial sensitivity patterns of the specific isolates. Our research has brought us to the conclusion that Ciprofloxacin is not to be recommended for empirical treatment in DFIs as it was particularly inactive against many strains of pathogens identified in the infections. Furthermore, initiation of treatment with broad-spectrum antimicrobials including Carbapenems and Piperacillin-Tazobactam for more-extensive chronic moderate and severe infections appears to be a safe measure. Definitive treatment can subsequently be initiated upon confirmation of offending pathogen via culture and sensitivity testing, susceptibility data and the patient’s response to empirical therapy from a clinical aspect. In order to implement targeted and correct antimicrobial therapy, it is essential to have knowledge and awareness of the common offending pathogens in DFIs.

The management of DFIs often calls for combination therapy with surgical intervention in the form of drainage and debridement or osseous resection especially if osteomyelitis was present [26]. In recent studies, recommendations have included treatment with Hyperbaric Oxygen Therapy (HBOT) as an adjunctive treatment in managing chronic DFIs as they can accelerate wound healing and improve patients’ quality of life [27]. The preference of antibiotic therapy is guided by the sensitivity of the bacterial pathogens encountered. Empirical antimicrobial therapy should utilise versatile antibiotics that have a wide coverage including the coverage of Gram-negative microorganisms, Gram-positive microorganisms and anaerobes if they are particularly suspected in infected foot ulcers. Detailed and meticulous microbiological workup is imperative to the selection of proper antibiotics for DFIs. Numerous drugs have been used to treat non-limb-threatening infections including Aminoglycosides, Ampicillin, Penicillin, Beta-lactamase inhibitors, third-generation Cephalosporins, Quinolones, Piperacillin-Tazobactam and Linezolid [13]. It was also found that Enterococci and anaerobes were not responsive to third-generation Cephalosporins, while Streptococci and anaerobes were said to have been less responsive to Fluoroquinolones [5, 28]. The limitation for this study is that the validated diabetic foot infection tool such as Infectious Diseases Society of America/International Working Group (IDSA/IWGDF) classification was not used as it is not been practiced routinely in our centre. Despite that, there is no difference in the management of our diabetic foot infections in compare to the practice that has been published elsewhere.

## Conclusion

The common aetiology of the DFIs in our patients was polymicrobial in nature in which P. Aeruginosa and S. Aureus were identified to be the most common agents and there was a low proportion of anaerobic organisms. Vancomycin, Piperacillin–Tazobactam or Imipenem appear to be suitable agents for empirical therapy according to antimicrobial susceptibility data. Clinicians are also encouraged to obtain adequate specimens after wound debridement for proper culture in addition to requesting for prompt microbiological reporting of all organisms which are obtained from the specimens. Considering the limited suitability of antibiotics, determination of the appropriate antimicrobial treatment should be guided by clinical correlation in addition to the local pattern of bacterial aetiology and its sensitivity. On the grounds that DFI’s are of polymicrobial aetiology, it is unlikely for a single antimicrobial agent to be effective against all the probable organisms isolated from diabetic foot infections. The findings of this study further emphasise the need to select antimicrobial treatment should be guided by proven culture results and antimicrobial sensitivity patterns exhibited by isolates. Choosing empirical antibiotic therapy will be guided by many factors including overall patient condition, glycaemic control, the severity of infection, allergies towards medication, prior antibiotic usage, antibiotic activity, excretion and toxicity. It is imperative to identify causative agents, appropriate antibiotic therapy and management of complications of diabetic foot infections in order to accomplish optimal results.

## Data Availability

All data generated or analysed during this study are included in this published article.

## Abbreviations

DFIs: Diabetic foot infections
MDROs: Multidrug-resistant organisms
CLSI: Clinical and Laboratory Standards Institute
DM: Diabetes Mellitus
WHO: World Health Organization
ESBLs: Extended spectrum beta-lactamases
MRSA: Methicillin-resistant *Staphylococcus aureus*
HBOT: Hyperbaric Oxygen Therapy
BA: Blood agar
API: Analytical profile index
SPP: Species
IDSA: Infectious Diseases Society of America
IWGDF: International Working Group of Diabetic Foot
